# Supplementary material for: The COX-2/PGE2 pathway suppresses apical elimination of RasV12-transformed cells from epithelia
Source: Commun Biol. 2020 Mar 18;3:132. doi: 10.1038/s42003-020-0847-y (PMC7080752; doi:10.1038/s42003-020-0847-y)
Supplement: Supplementary file 1 — Supplementary Information [file 42003_2020_847_MOESM1_ESM.pdf]

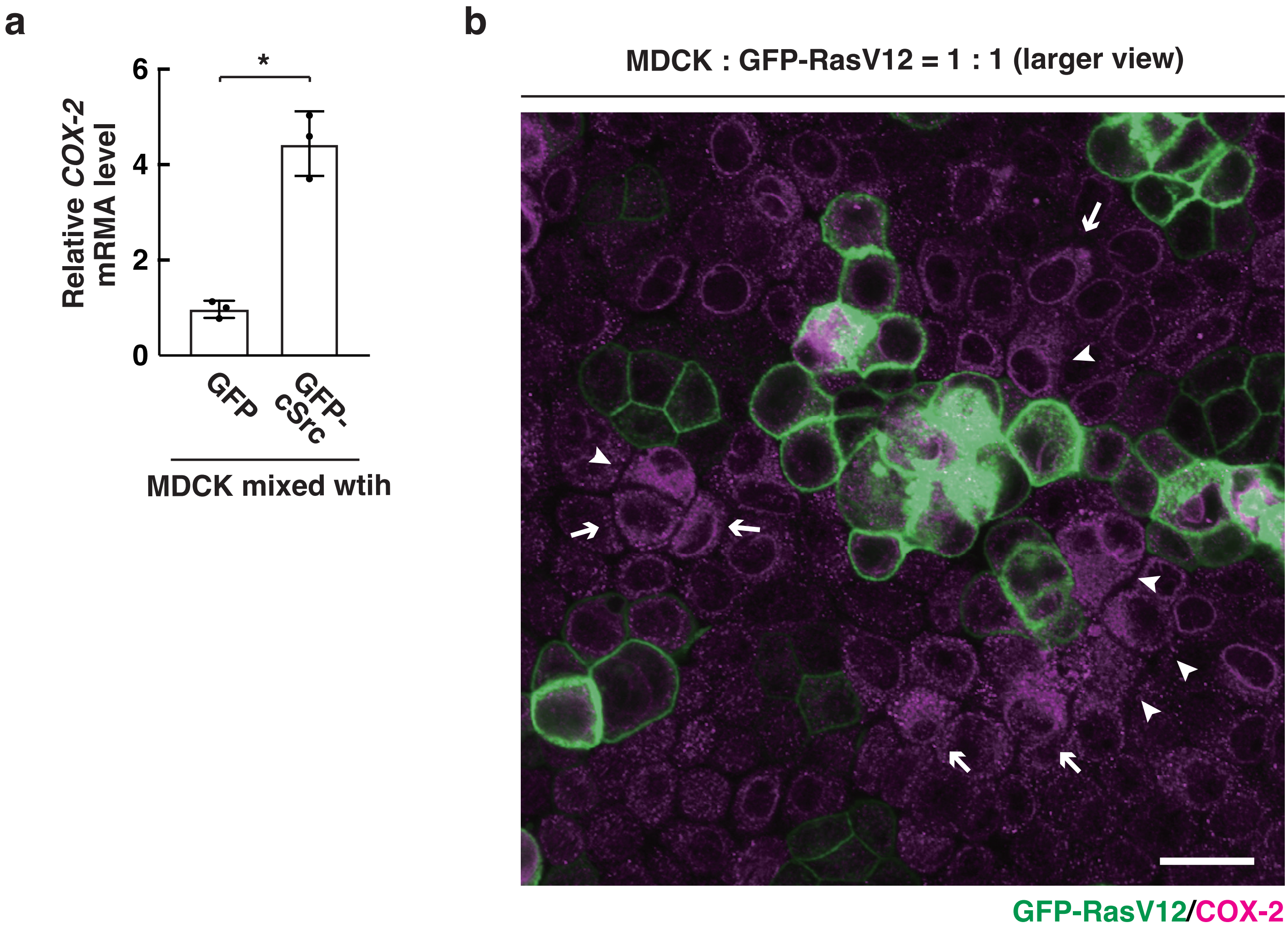

Supplementary Fig. 1 a Quantitative RT-PCR analysis of COX-2 expression in normal MDCK cells co-cultured with GFP- or GFP-cSrcY527F-expressing MDCK cells. Cell lysates from FACS-sorted GFP-negative normal cells were examined. Data are mean ± s.d. from three independent experiments. Values are expressed as a ratio relative to GFP. **P*=0.0088 (Student’ s *t*-test). **b** Immunofluorescence analysis of COX-2 expression. MDCK cells were co-cultured with MDCK GFP-RasV12 cells at a ratio of 1:1, followed by immunofluorescence analysis with anti-COX-2 antibody. Arrowheads or arrows indicate COX-2 positive cells directly contacting or not contacting RasV12 cells, Note that the upregulation of COX-2 expression was often observed within two cell-rows from RasV12 cells. Scale bars, 20 µm.

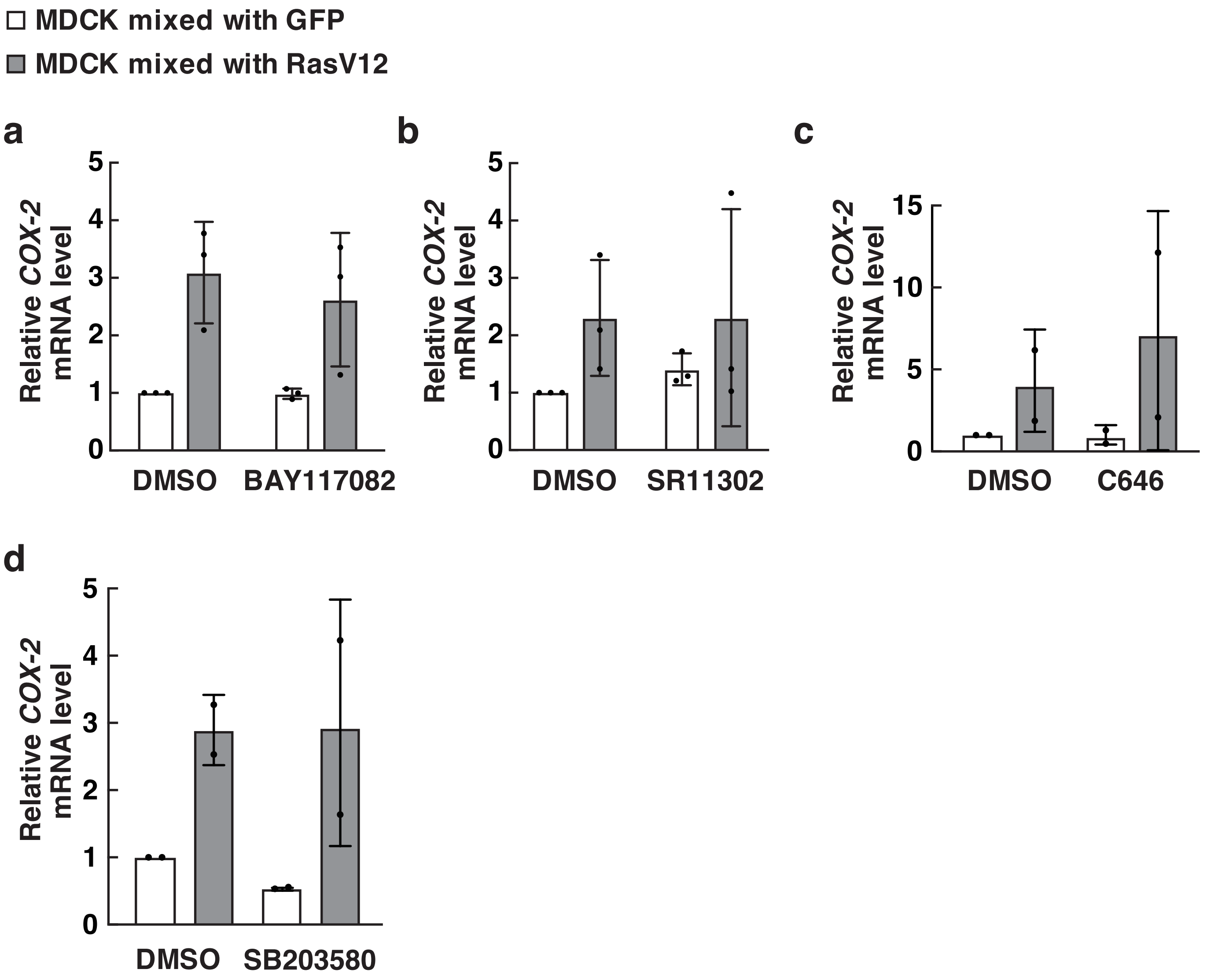

Supplementary Fig. 2 Effect of various inhibitors on the COX-2 mRNA level in normal MDCK cells co-cultured with GFP-expressing MDCK cells (white) or GFP-RasV12-expressing MDCK cells (grey). Data are mean ± s.d. from three (a and b) or two (c and d) independent experiments. Values are expressed as a ratio relative to DMSO (MDCK mixed with GFP). (a) BAY117082: NF-κB inhibitor, (b) SR11302: AP-1 inhibitor, (c) C646: CREB inhibitor, (d) SB203580: p38MAPK inhibitor.

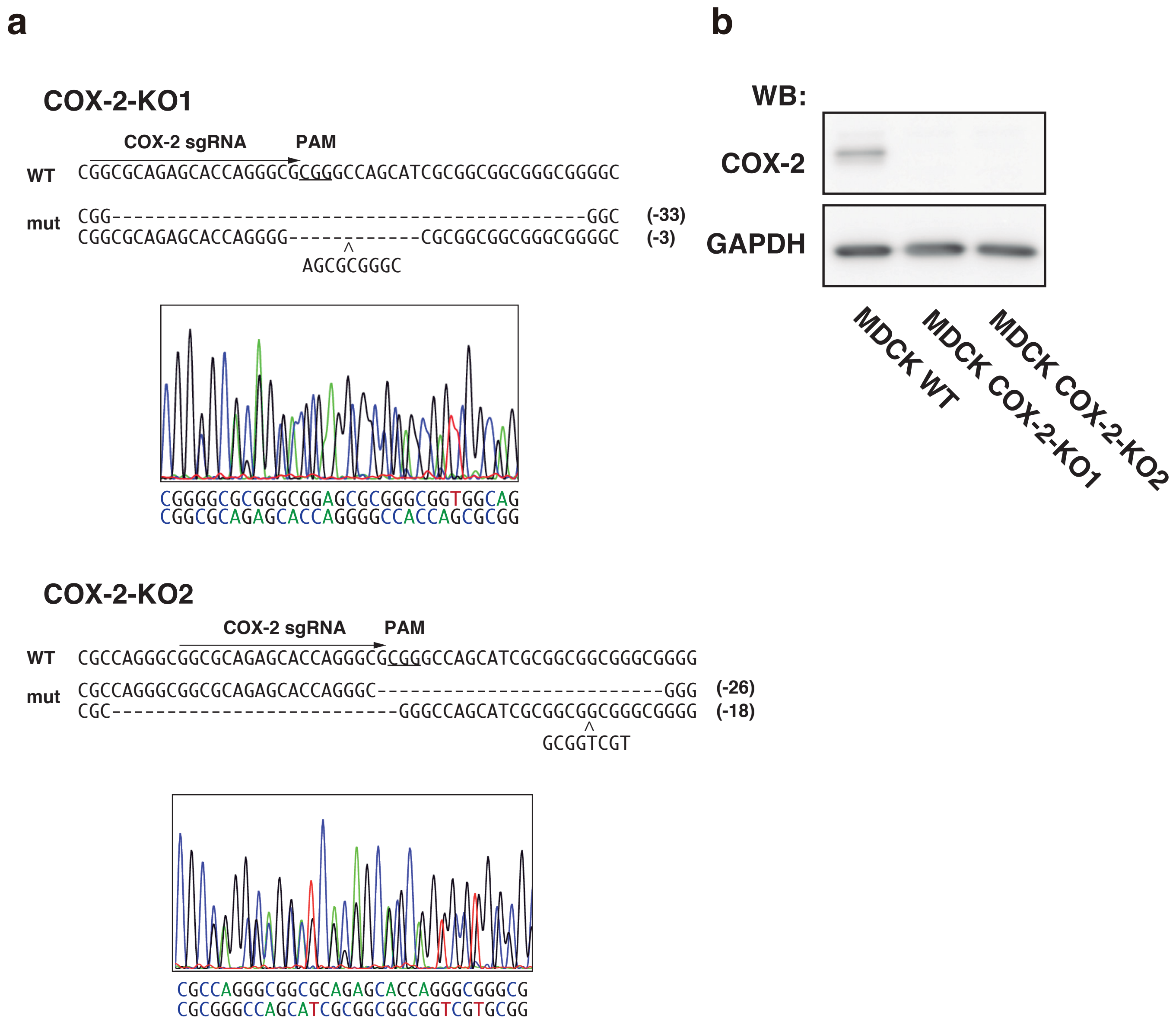

Supplementary Fig. 3 Establishment of COX-2-knockout MDCK cell lines. **a** A targeting scheme and DNA sequences of the wild type and COX-2-null MDCK cell lines. PAM motifs are underlined. The spacing indicates a deleted nucleotide. **b** Knockout of COX-2 was confirmed by western blotting. Note that RasV12 was stably expressed in MDCK COX-2-KO1 cells to establish the RasV12 COX-2-knockout cell line.

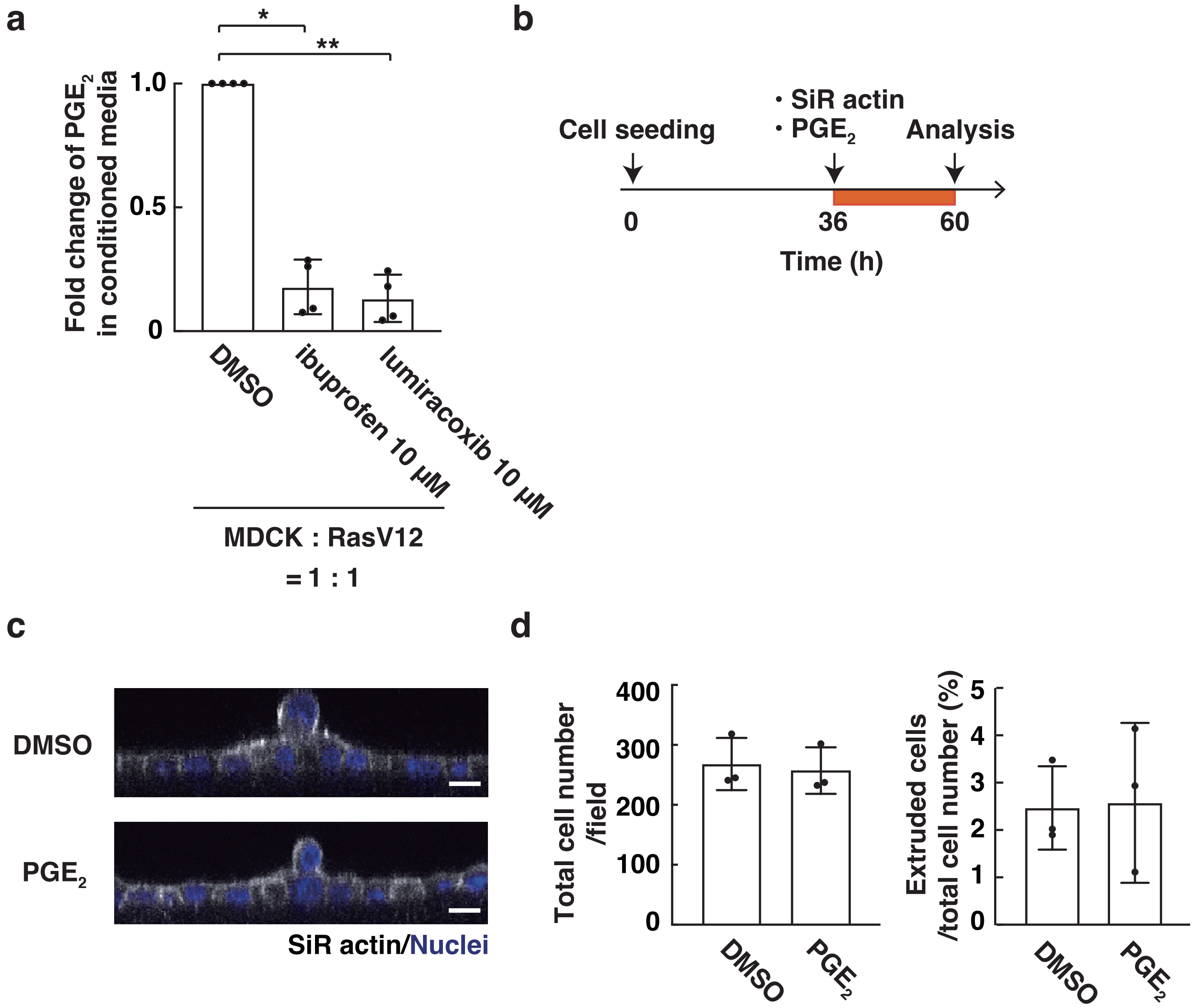

Supplementary Fig. 4

a Effect of the COX inhibitor on the PGE_2 level in the conditioned medium of the mix culture of normal and RasV12-transformed cells. Data are mean ± s.d. from four independent experiments. Values are expressed as a ratio relative to DMSO. $^{*}P=6.6 \times 10^{-4}$, $^{**}P=3.6 \times 10^{-4}$ (Student' s *t*-test). **b** Experimental design for analyses of crowded cell extrusion. **c** The xz-immunofluorescence images of extruded cells from the epithelial layer in the absence or presence of PGE_2 at a high cell density. To stain F-actin, we used SiR actin probe to prevent wash-out of extruded cells. Scale bars, 20 μm. **d** Effect of PGE_2 on cell number and extrusion. Data are mean ± s.d. from three independent experiments. Note that PGE_2 treatment did not significantly influence cell growth.

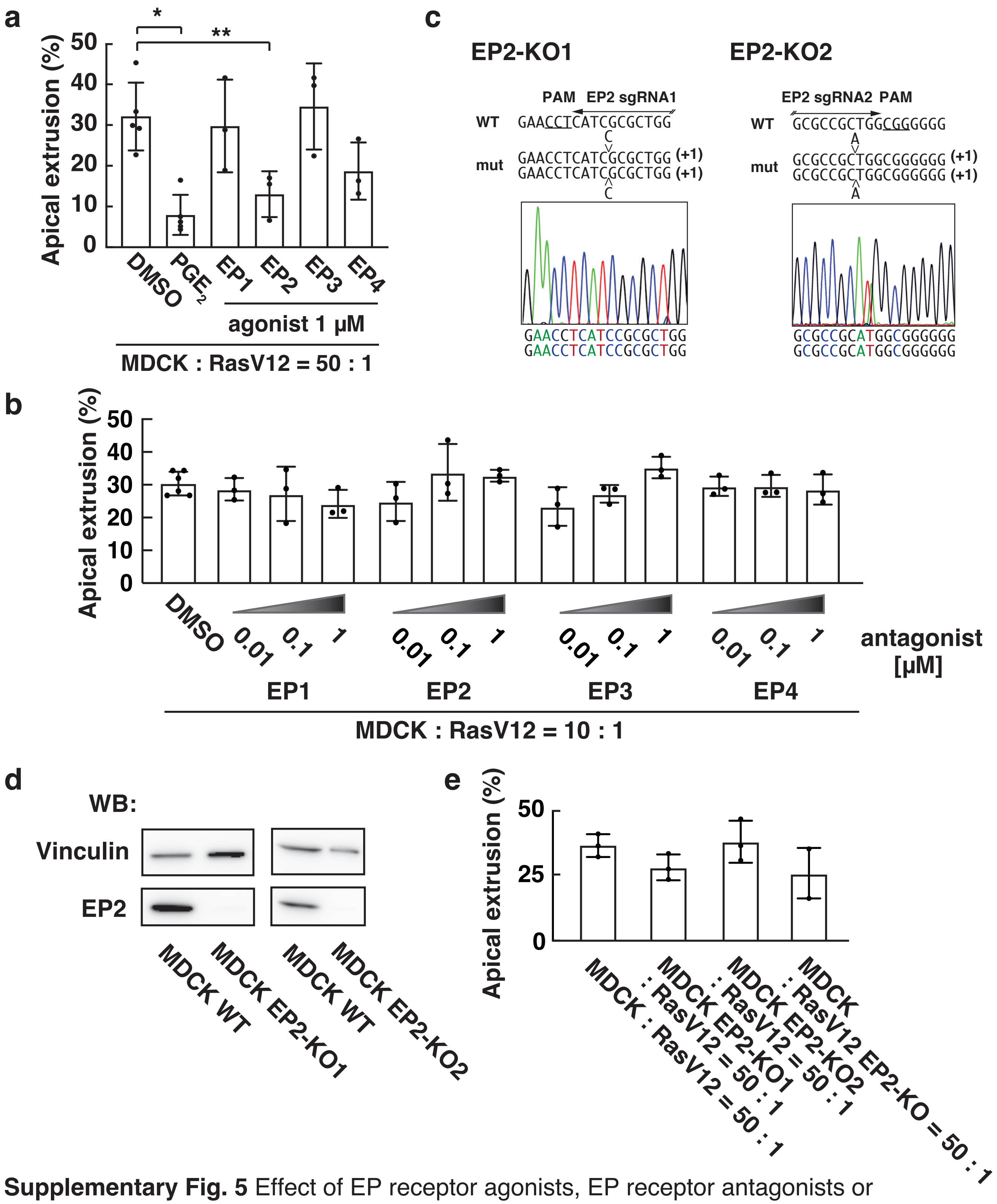

Supplementary Fig. 5 Effect of EP receptor agonists, EP receptor antagonists or EP2-knockout on apical extrusion of RasV12-transformed cells. **a** Effect of PGE_2 (0.1 μM) or EP receptor agonists on apical extrusion of RasV12 cells. Data are mean ± s.d. from six (DMSO and PGE_2) or three (EP1-EP4) independent experiments. **P*=0.0011, ***P*=0.0091 (unpaired t-test). **b** Effect of EP receptor antagonists on apical extrusion of RasV12 cells. Data are mean ± s.d. from six (DMSO) or three (EP1-EP4) independent experiments. **c** A targeting scheme and DNA sequences of the wild type and EP2-null MDCK cell lines. PAM motifs are underlined. **d** Knockout of EP2 was confirmed by western blotting. Note that RasV12 was stably expressed in MDCK EP2-KO2 cells to establish the RasV12 EP2-knockout cell line. **e** Effect of EP2-knockout in normal cells or RasV12 cells on apical extrusion of RasV12 cells. Data are mean ± s.d. from three (left three) or two (right) independent experiments.

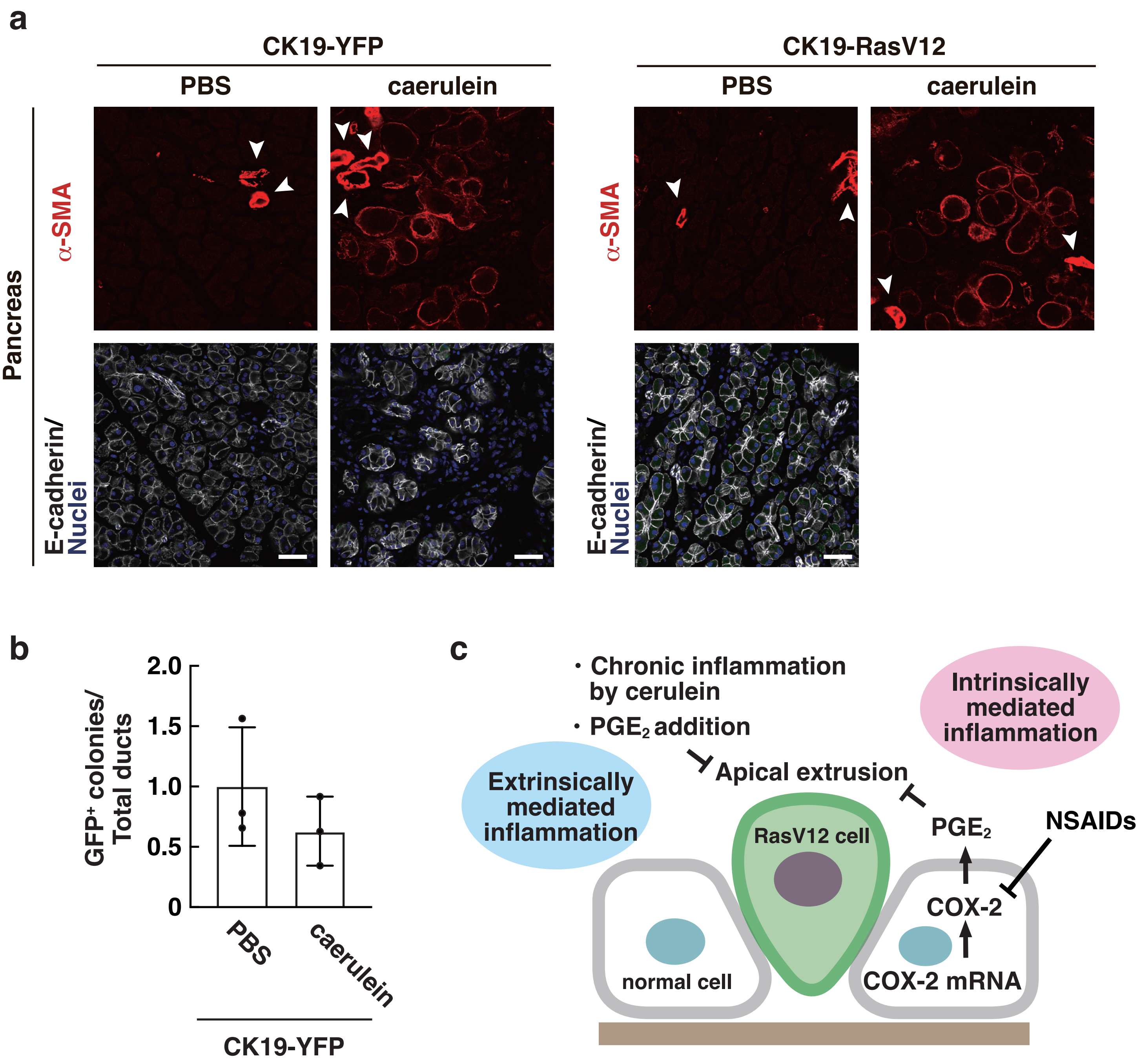

Supplementary Fig. 6

a Immunofluorescence analysis for the active pancreatic stellate cell marker α-SMA in the pancreatic tissues upon caerulein treatment. Arrowheads indicate α-SMA-positive smooth muscle cells around blood vessels. Scale bars, 40 μm. **b** Quantification of YFP-expressing cell groups (colonies) in the pancreatic epithelial ducts in the presence or absence of caerulein treatment. The total number of analysed ducts are 261 (PBS) and 377 (caerulein). Values are expressed as a ratio relative to PBS. Data are mean ± s.d. from three mice. **c** Schematics for the inhibitory role of inflammation in apical extrusion of RasV12-transformed cells.

	Name	5' to 3' sequence
Site-directed mutagenesis primer	canis sgCOX-2 QC primer Fw	GGAAAGGACGAAACACCGTAGGATTAACAGGCTTTAACGTTTTAGAGCTAGAAATAGC
	canis sgCOX-2 QC primer Rv	GCTATTTCTAGCTCTAAAACGTTAAAGCCTGTTAATCCTACGGTGTTTCGTCCTTTCC
	canis sgEP2#1 QC primer Fw	GGAAAGGACGAAACACCGCAGCAGCGCCAGCGCGATGGTTTTAGAGCTAGAAATAGC
	canis sgEP2#1 QC primer Rv	GCTATTTCTAGCTCTAAAACCATCGCGCTGGCGCTGCTGCGGTGTTTCGTCCTTTCC
	canis sgEP2#2 QC primer Fw	GGAAAGGACGAAACACCGGCTGCTGGCGCGCCGCTGGGTTTTAGAGCTAGAAATAGC
	canis sgEP2#2 QC primer Rv	GCTATTTCTAGCTCTAAAACCCAGCGGCGCGCCAGCAGCCGGTGTTTCGTCCTTTCC
Primers used for direct sequencing	canis COX-2 KO ex1 Fw	GGAAGGTTCCGTCCGTTAG
	canis COX-2 KO ex1 Rv	ATGACAAGGGAGACCCACAC
	canis EP2 KO ex1 Fw	CCTGCTCCCAGACTCTCCT
	canis EP2 KO ex1 Rv	GAGGCTGAAGAAGGTCATGG
genotyping primer	CK19-CreERT2 Fw	AATCGCCAGGAATTGACCAATGGGG
	CK19-CreERT2 Rv	CGCCCGTACCCCCAAAGGAAGACAT
	DNMT1-CAG-loxP-STOP-loxP-HRasV12-IRES-eGFP Fw	CACTGTGGAATCTCGGCAGG
	DNMT1-CAG-loxP-STOP-loxP-HRasV12-IRES-eGFP Rv	GCAATATGGTGGAAAATAAC
	LSL-YFP Fw	AAAGTCGCTCTGAGTTGTTAT
	LSL-YFP mut Rv	GGAGCGGGAGAAATGGATATG
	LSL-YFP wt Rv	GGAGCGGGAGAAATGGATATG
qPCR primer	canis PTGS2 Fw	CGAGGACCAGCTTTCACCA
	canis PTGS2 Rv	GACCTGAGTATCTTTGACGGTAGGA
	canis GAPDH Fw	ATTCTATCCACGGCAAATCC
	canis GAPDH Rv	GGACTCCACAACATACTAG
	mouse TNF alpha Fw	CTGTAGCCCACGTCGTAGC
	mouse TNF alpha Rv	TTGAGATCCATGCCGTTG
	mouse IL-1 beta Fw	TTGACGGACCCCAAAAGAT
	mouse IL-1 beta Rv	GAAGCTGGATGCTCTCATCTG
	mouse CCL2 Fw	CATCCACGTGTTGGCTCA
	mouse CCL2 Rv	GATCATCTTGCTGGTGAATGAGT
	mouse IL-6 Fw	TGCCTTCATTTATCCCTTGAA
	mouse IL-6 Rv	TTACTACATTCAGCCAAAAAGCAC
	mouse RPL13A Fw	GAGGTCGGGTGGAAGTACCA
	mouse RPL13A Rv	TGCATCTTGGCCTTTTCCTT
	mouse PTGS2 Fw	GATGCTCTTCCGAGCTGTG
	mouse PTGS2 Rv	GGATTGGAACAGCAAGGATTT

Supplementary Table 1

Primer sequences for site-directed mutagenesis, direct sequencing, genotyping and qPCR
