# Supplementary material for: The COX-2/PGE2 pathway suppresses apical elimination of RasV12-transformed cells from epithelia
Source: Commun Biol. 2020 Mar 18;3:132. doi: 10.1038/s42003-020-0847-y (PMC7080752; doi:10.1038/s42003-020-0847-y)
Supplement: Supplementary file 2 — Description of additional supplementary files [file 42003_2020_847_MOESM2_ESM.pdf]

Description of additional supplementary files

Supplementary Data 1

Excel file showing the source data behind the graphs
