# Supplementary material for: The COX-2/PGE2 pathway suppresses apical elimination of RasV12-transformed cells from epithelia
Source: Commun Biol. 2020 Mar 18;3:132. doi: 10.1038/s42003-020-0847-y (PMC7080752; doi:10.1038/s42003-020-0847-y)
Supplement: Supplementary file 5 — Reporting Summary [file 42003_2020_847_MOESM5_ESM.pdf]

nature research

Corresponding author(s): Yasuyuki Fujita

Last updated by author(s): Jan 4, 2020

Reporting Summary

Nature Research wishes to improve the reproducibility of the work that we publish. This form provides structure for consistency and transparency in reporting. For further information on Nature Research policies, see Authors & Referees and the Editorial Policy Checklist.

Statistics

For all statistical analyses, confirm that the following items are present in the figure legend, table legend, main text, or Methods section.

n/a	Confirmed	
☐	☒	The exact sample size (*n*) for each experimental group/condition, given as a discrete number and unit of measurement
☐	☒	A statement on whether measurements were taken from distinct samples or whether the same sample was measured repeatedly
☐	☒	The statistical test(s) used AND whether they are one- or two-sided<br>*Only common tests should be described solely by name; describe more complex techniques in the Methods section.*
☒	☐	A description of all covariates tested
☒	☐	A description of any assumptions or corrections, such as tests of normality and adjustment for multiple comparisons
☐	☒	A full description of the statistical parameters including central tendency (e.g. means) or other basic estimates (e.g. regression coefficient) AND variation (e.g. standard deviation) or associated estimates of uncertainty (e.g. confidence intervals)
☒	☐	For null hypothesis testing, the test statistic (e.g. *F*, *t*, *r*) with confidence intervals, effect sizes, degrees of freedom and *P* value noted<br>*Give P values as exact values whenever suitable.*
☒	☐	For Bayesian analysis, information on the choice of priors and Markov chain Monte Carlo settings
☒	☐	For hierarchical and complex designs, identification of the appropriate level for tests and full reporting of outcomes
☒	☐	Estimates of effect sizes (e.g. Cohen's *d*, Pearson's *r*), indicating how they were calculated

Our web collection on statistics for biologists contains articles on many of the points above.

Software and code

Policy information about availability of computer code

Data collection	No software was used.
Data analysis	PRISM7 was used for Mann-Whitney test.

For manuscripts utilizing custom algorithms or software that are central to the research but not yet described in published literature, software must be made available to editors/reviewers. We strongly encourage code deposition in a community repository (e.g. GitHub). See the Nature Research guidelines for submitting code & software for further information.

Data

Policy information about availability of data

All manuscripts must include a data availability statement. This statement should provide the following information, where applicable:

- Accession codes, unique identifiers, or web links for publicly available datasets
- A list of figures that have associated raw data
- A description of any restrictions on data availability

The data that support the findings of this study are available from the corresponding author upon reasonable request.

Field-specific reporting

Please select the one below that is the best fit for your research. If you are not sure, read the appropriate sections before making your selection.

☒ Life sciences ☐ Behavioural & social sciences ☐ Ecological, evolutionary & environmental sciences

For a reference copy of the document with all sections, see nature.com/documents/nr-reporting-summary-flat.pdf

Life sciences study design

All studies must disclose on these points even when the disclosure is negative.

Sample size	The sample size is described in Figure legends for each experiment. No statical methods were used to predetermine sample size.
Data exclusions	N/A
Replication	All experiments are repeated at least twice (more than three times in most cases), and the results were well reproduced.
Randomization	No randomization was used.
Blinding	No blinding was done.

Reporting for specific materials, systems and methods

We require information from authors about some types of materials, experimental systems and methods used in many studies. Here, indicate whether each material, system or method listed is relevant to your study. If you are not sure if a list item applies to your research, read the appropriate section before selecting a response.

Materials & experimental systems

n/a	Involved in the study	
☐	☒	Antibodies
☐	☒	Eukaryotic cell lines
☒	☐	Palaeontology
☐	☒	Animals and other organisms
☒	☐	Human research participants
☒	☐	Clinical data

Methods

n/a	Involved in the study	
☒	☐	ChIP-seq
☐	☒	Flow cytometry
☒	☐	MRI-based neuroimaging

Antibodies

Antibodies used

Antibody (Cat #) Supplier
mouse anti-beta-actin (MAB1501R clone C4) Merck Millipore
mouse anti-GAPDH (MAB374) Merck Millipore
mouse anti-filamin (F6682) Sigma-Aldrich
mouse anti-vinculin (V44505) Sigma-Aldrich
rabbit anti-COX-2 (ab15191) Abcam
chicken anti-GFP (ab13970) Abcam
rabbit anti-Phospho-(Ser) PKC Substrate (#2261) Cell Signaling
rat anti-E-cadherin (1900) Life Technologies
α-Smooth Muscle - Cy3™ antibody (C6198) Sigma-Aldrich
APC-conjugated CD45.2 (20-0454) TONBO

Validation

Some of the antibodies were validated by testing the effect of knockdown of the epitope molecule. Otherwise, the antibodies have been validated by the other groups.

Eukaryotic cell lines

Policy information about cell lines

Cell line source(s)	MDCK cells were a gift from W. Birchmeier (Fujita et al., 2002)
Authentication	The cell line used in this study was not authenticated.
Mycoplasma contamination	In our lab, mycoplasma contamination is regularly tested for all cell lines in use using a commercially available kit (MycoAlert, Lonza).
Commonly misidentified lines (See ICLAC register)	N/A

Animals and other organisms

Policy information about studies involving animals; ARRIVE guidelines recommended for reporting animal research

Laboratory animals	We used 8-36weeks old C57BL/6 mice of either sex.
Wild animals	This study did not involve wild animals.
Field-collected samples	N/A
Ethics oversight	All animal experiments were conducted under the guidelines by the Animal Care Committee of Hokkaido University. The animal protocols were reviewed and approved by the Hokkaido University Animal Care Committee (Approval number:12-0116), which is described in the Method section.

Note that full information on the approval of the study protocol must also be provided in the manuscript.

Flow Cytometry

Plots

Confirm that:

☒ The axis labels state the marker and fluorochrome used (e.g. CD4-FITC).

☒ The axis scales are clearly visible. Include numbers along axes only for bottom left plot of group (a 'group' is an analysis of identical markers).

☒ All plots are contour plots with outliers or pseudocolor plots.

☒ A numerical value for number of cells or percentage (with statistics) is provided.

Methodology

Sample preparation	Details are described in Method.
Instrument	FACS Aria II
Software	FACSDiva for cell collection
Cell population abundance	0.5 million GFP-cells were sorted.
Gating strategy	Details are described in Method.

☐ Tick this box to confirm that a figure exemplifying the gating strategy is provided in the Supplementary Information.
